# Multiple-platform data integration method with application to combined analysis of microarray and proteomic data

**DOI:** 10.1186/1471-2105-13-320

**Published:** 2012-12-02

**Authors:** Shicheng Wu, Yawen Xu, Zeny Feng, Xiaojian Yang, Xiaogang Wang, Xin Gao

**Affiliations:** 1 Department of Mathematics and Statistics, York University, 4700 Keele, Street, Toronto, Ontario, M3J 1P3 Canada; 2 Department of Mathematics and Statistics, 50 Stone Road East, Guelph, Ontario, N1G 2W1 Canada

## Abstract

**Background:**

It is desirable in genomic studies to select biomarkers that differentiate between normal and diseased populations based on related data sets from different platforms, including microarray expression and proteomic data. Most recently developed integration methods focus on correlation analyses between gene and protein expression profiles. The correlation methods select biomarkers with concordant behavior across two platforms but do not directly select differentially expressed biomarkers. Other integration methods have been proposed to combine statistical evidence in terms of ranks and p-values, but they do not account for the dependency relationships among the data across platforms.

**Results:**

In this paper, we propose an integration method to perform hypothesis testing and biomarkers selection based on multi-platform data sets observed from normal and diseased populations. The types of test statistics can vary across the platforms and their marginal distributions can be different. The observed test statistics are aggregated across different data platforms in a weighted scheme, where the weights take into account different variabilities possessed by test statistics. The overall decision is based on the empirical distribution of the aggregated statistic obtained through random permutations.

**Conclusion:**

In both simulation studies and real biological data analyses, our proposed method of multi-platform integration has better control over false discovery rates and higher positive selection rates than the uncombined method. The proposed method is also shown to be more powerful than rank aggregation method.

## Background

In gene expression experiments, the expression levels of thousands of genes are simultaneously monitored to study the underlying biological process. In proteomic data, the protein levels or protein counts are measured for thousands of genes simultaneously. In addition, there are other types of genomic data with different sizes, formats and structures. Each distinct data type, such as gene expression, protein counts, or single nucleotide polymorphisms, provide potentially valuable and complementary information regarding the involvement of a given gene in a biological process. Many biomarkers that play important roles in biological processes behave differently in treatment versus control groups; this phenomenon can be observed consistently across various data platforms. Therefore, integrating related data sets from different sources is crucial to correctly identify the significant underlying biomarkers. Integrative analysis of multiple data types would improve the identification of biomarkers of clinical end points [1]. However, the integration of data from different sources poses a number of challenges. First, genomic data come in a wide variety of data formats. For example, expression data are recorded as continuous measurements, whereas proteomic data often consist of discrete counting variables. One may wish to convert data into a common format and common dimension, but this is not always practical or feasible [2]. Second, different data sets are collected under different experimental settings. Therefore, the distribution of the measurements as well as the quality of the experiments may vary from data set to data set. Third, measurements obtained across different data platforms could be collected from the same or related biological samples. Therefore, measurements across different data types could have complicated dependency relationships.

The practice of combining different data sources to perform classification analysis has been considered in the literature. Efforts to integrate data and improve classification accuracy are widely seen in recent studies [3-5]. In contrast to performing classification on biological samples, our main objective is to select important biomarkers for an underlying biological process. Correlation analysis has been proposed to integrate diverse data types and assimilate them into biological models for the prediction of cellular behavior and clinical outcome. Tian et al. [6] performed a correlation analysis of protein and mRNA expression data using the cosine correlation metric for comparison. Bussey et al. [7] integrated data on DNA copy number with gene expression levels and drug sensitivities in cancer cell lines based on Pearson’s correlation coefficients. Adourian et al. [8] presented a cross-compartment correlation network approach to integrate proteomic, metabolomic, and transcriptomic data for selecting circulating biomarkers; partial pairwise Pearson’s correlations controlling for treatment group means were calculated. The markers with concordant RNA and protein expression were included in the prediction models, while discordant ones were excluded. However, this approach might miss some important biological information, such as protein-protein interactions and protein-gene interactions [9]. Another limitation is that correlation analysis mainly captures the strength of the correlation among measurements across different platforms; however, strong correlation only demonstrates consistent outcome across different platforms and does not directly translate to significant involvement in a biological process. Furthermore, statistical evidence from complicated data sets, such as factorial experiments, times series, or longitudinal data, cannot be summarized.

The problem of how to reliably combine data from different experiment platforms to identify significant biomarkers has recently received considerable attention in the bioinformatics literature. The rank aggregation method [10] has been proposed for ranking genes by similarity to the disease genes in Gene Ontology, pathways, transcription factor binding sites, and sequence, then aggregating this rankings to get the final result. Rhodes et al. [11] combined four independent data sets to identify genes deregulated in prostate cancer. For each gene in each data set, a p-value was obtained as an indication of the probability that the gene was differentially expressed. P-values for different data sets were subsequently aggregated to provide an overall estimate of the genes’ significance of being differentially expressed during prostate cancer. However, combining genes’ ranks in the rank aggregation approach or p-values in the meta-profiling method ignores the underlying multivariate distributions of the ranks or p-values. Furthermore, data quality may vary across different data sources. The two aggregation methods detailed above essentially give equal weights to different data sets. Thus, we propose to combine statistical evidence across different platforms through summary statistics instead of raw data. For each experimental platform, we formulate a null hypothesis and construct the summary test statistic. By randomization, we obtain the null distribution of the vector of statistics across different platforms. The test statistics are summarized across different platforms in a weighted scheme, where the weights take into account different variabilities possessed by the statistics. The method allows the use of different types of summary statistics from different platforms, which gives great flexibility and generality with respect to its application.

The proposed method is similar in spirit to a meta-analysis. Both methods combine statistical evidence across multiple data sets. However, in meta-analysis different data sets are based on the same type of experiments or observational studies, and therefore the measurements are the same variables. Across different data sets, the quality of the data may vary. The goal of meta-analysis is to fully utilize all the information from different data sets and construct a weighted estimate of the effect size. Different weighting schemes are available depending on the statistical models [12]. On the other hand, data integration focuses on integrating statistical evidence across different experimental types. There is no common effect size to estimate across various data sets. In our proposed method, we use a weighted average of the test statistics across different data platforms, but the test statistics are summaries of evidence towards different sub-hypotheses rather than summaries of common effect size as in meta-analysis. The proposed integration method does not check for differences across the platforms.

## Methods

The aim of our multi-platform integration method is to select a set of significant biomarkers that are involved in a biological process and thus behave differently in the treatment group and the control group. In order to combine statistical evidence across different platforms, our method requires that analogous hypotheses based on the features being measured are formulated for each platform. Each null analogous hypothesis specifies the unrelatedness of the biomarker in that particular experimental setting, but all of them infer the unrelatedness of the biomarker to the biological process being investigated. Based on the set of Q analogous hypotheses for Q data sources, we construct a set of Q corresponding test statistics for each type of data. The test statistics can be different and tailored to the specific experimental settings. For example, if the microarray experiment has a multifactorial design, the appropriate test statistic can be an F statistic based on an ANOVA test. If the proteomics experiment generates counting data for diseased versus normal groups, the appropriate test statistic can be a nonparametric Wilcoxon rank sum test. A vector of observed statistics across multi-platforms is obtained. We then randomly permute data across diseased and control groups. All measurements from different platforms are permuted. In this way, we obtain an empirical null distribution of the vector of test statistics. In order to pool the randomized values of the statistics across the biomarkers to form the empirical null distribution, we assume data from different biomarkers are independent or have an exchangeable correlation structure. For the validity of the randomization procedure, we assume an exchangeable covariance structure for the measurements within each platform. Finally, we construct a weighted sum of the test statistics across different platforms with the weights being the inverse of the empirical standard deviation of each statistic. We determine a set of significant biomarkers based on the aggregated test statistic.

In the following, we demonstrate our method by integrating microarray expression data and proteomic data as an example. We consider two experiments, the first having microarray expression data measured on *l*_1_ diseased samples and *l*_2_ control samples and the second having proteomic data measured on *m*_1_ diseases samples and *m*_2_ control samples. The objective is to find biomarkers significantly involved in disease development. 

Step 1): Define two analogous null hypotheses. For microarray data, the null hypothesis would be *H*_01_: the gene’s mRNA level is the same in diseased and normal populations; for proteomic data, the null hypothesis would be *H*_02_: the protein level is the same in diseased and normal populations.

Step 2): Based on the hypotheses, construct two test statistics, *t*_*m*_ and *t*_*p*_, tailored to each type of data. Consequently, we obtain a vector of two observed statistics (*t*_*m*_,*t*_*p*_)^*′*^ across two data platforms. The test statistics can be of any type as long as they summarize information from the data and can be used to assess the statistical significance of the data toward the hypotheses. Let $x_{1}={\left(x_{11},\ldots ,x_{1l_{1}}\right)}^{\prime }$ denote the *l*_1_ gene expression measurements in the disease group, $x_{2}={\left(x_{21},\ldots ,x_{2l_{2}}\right)}^{\prime }$ denote the *l*_2_ gene expression measurements in the control group, ${\bar{x}}_{1}=\sum_{j=1}^{l1}x_{1j}/l_{1}$, and ${\bar{x}}_{2}=\sum_{j=1}^{l2}x_{2j}/l_{2}$. Similarly, $y_{1}={\left(y_{11},\ldots ,y_{1m_{1}}\right)}^{\prime }$ denotes the *m*_1_ protein measurements in the disease group and $y_{2}={\left(y_{21},\ldots ,y_{2m_{2}}\right)}^{\prime }$ denotes the *m*_2_ protein measurements in the control group, ${\bar{y}}_{1}=\sum_{j=1}^{m_{1}}y_{1j}/m_{1}$, and ${\bar{y}}_{2}=\sum_{j=1}^{m_{2}}y_{2j}/m_{2}$. For illustration purpose, we adopt Student’s t-statistic for each of the data:

$$t_{m}=\frac{{\bar{x}}_{2}-{\bar{x}}_{1}}{\sqrt{\frac{s^{2}\left(x_{1}\right)}{l_{1}}+\frac{s^{2}\left(x_{2}\right)}{l_{2}}}},$$

and

$$t_{p}=\frac{{\bar{y}}_{2}-{\bar{y}}_{1}}{\sqrt{\frac{s^{2}\left(y_{1}\right)}{m_{1}}+\frac{s^{2}\left(y_{2}\right)}{m_{2}}}},$$

where *s*^2^ denotes the sample variance. The test statistics should be formulated so that a larger test statistic in the positive direction indicates more evidence towards the alternative hypotheses. For example, if Student’s t-statistic is used, then a one-sided alternative hypothesis corresponds to a one-sided t-statistic, whereas the two-sided alternative leads to the absolute value of the t-statistic. Consider *n* genes being measured in the experiments and we obtain *n* vectors of test statistics (*t*_*mi*_,*t*_*pi*_)^*′*^, *i* = 1,…,*n*, from the data sets.

Step 3): The samples are randomly permuted across diseased and control groups. If the same sample is being measured across different platforms, all the measurements from the different platform are permuted simultaneously. The simultaneous permutation preserves the dependency relationship among the measurements from different platforms. Based on random permutation, we obtain an empirical null distribution of the vector (*t*_*m*_,*t*_*p*_)^*′*^.

Step 4): The aggregated test statistic will be:

$$t_{A}=\frac{t_{m}}{{\overset{ˆ}{\sigma }}_{1}}+\frac{t_{p}}{{\hat{\sigma }}_{2}},$$

where ${\hat{\sigma }}_{1}$ and ${\hat{\sigma }}_{2}$ are the estimated standard deviations of *t*_*m*_ and *t*_*p*_ based on the empirical null distribution, and *t*_*m*_ and *t*_*p*_ are the observed t-statistics or the absolute values of the t-statistics based on the direction of the alternative hypotheses. At significance level *α*, we choose a threshold *C*_*α*_, such that $P_{H_{01}\cap H_{02}}\left(t_{A},>,C_{\alpha }\right)=\alpha$. Specifically, *C*_*α*_ is the 100(1−*α*)*%* percentile of *t*_*A*_, which can be obtained from the empirical null distribution. Construct a decision line that separates selected significant biomarkers and nonsignificant biomarkers. The resulting separation line is:

$$\frac{t_{m}}{{\hat{\sigma }}_{1}}+\frac{t_{p}}{{\hat{\sigma }}_{2}}=C_{\alpha }.$$

All the biomarkers with (*t*_*m*_,*t*_*p*_) above the separation line will be declared as significantly involved in the disease development.

In the more general case, suppose we have Q data platforms with the observed test statistics (*t*_1_,…,*t*_*Q*_)^*′*^. From random permutation, we obtain the joint empirical distribution of this vector of test statistics under the global null hypothesis. Let ${\hat{\sigma }}_{1}^{2},\ldots ,{\hat{\sigma }}_{Q}^{2}$ denote the estimated variance of the individual test statistics.The aggregated test statistic takes the form:

$$t_{A}=\overset{Q}{\underset{i=1}{\sum }}\frac{t_{i}}{{\hat{\sigma }}_{i}}.$$

The resulting critical region will take the form:

$$\frac{t_{1}}{{\hat{\sigma }}_{1}}+\ldots .+\frac{t_{Q}}{{\hat{\sigma }}_{Q}}>C_{\alpha },$$

where *C*_*α*_ is the 100(1−*α*)*%* percentile of *t*_*A*_. Any biomarker with *t*_*A*_ > *C*_*α*_ will be selected as behaving significantly differently between the diseased group and control group.

Our method aggregates actual values of the test statistics across different data platforms, which preserves more information compared to the rank aggregation method. Moreover, our method assigns different weights to each data set according to the variability of the test statistics: larger the variation in the test statistic, the smaller the weight assigned to it, and vice versa. The threshold *C*_*α*_ is determined based on the empirical null distribution of the aggregated test statistics, which implicitly takes into account the dependency relationships among the test statistics. Furthermore, our method can deal with different data types and formats generated by various experimental settings.

There are two major ways to perform the multiplicity adjustment. The first is the Bonferroni correction. If we wish to control the familywise type I error rate at *α*^∗^, then the individual level *α* = *α*^∗^/*n*, where *n* is the total number of biomarkers. When *n* is large, the Bonferroni correction leads to very stringent tests with *α* being very small. Alternatively, we can control the number of false discoveries. To set the number of false discoveries to be equal to or less than *f *, then $\alpha =f/\left(n\overset{ˆ}{\pi }\right)$, where $\hat{\pi }$ is the estimated proportion of non-differentially expressed biomarkers. If there is no $\hat{\pi }$ available, we use $\hat{\pi }=1$ and that gives a conservative value for *α*.

Different platforms can be used to test different sub-hypothesis. All of these sub-hypotheses should be concordant in supporting the overall biological hypothesis. For example, the involvement of a gene in disease development can be supported by both mRNA expression level changes and proteomic level changes. In most cases, changes in measurements from different platforms are expected to occur in the same direction. However, our method is also applicable even if the changes are in different directions, as long as the statistical evidence from both sources can be combined. For example, consider *H*_10_: mRNA is increasing in normal group; *H*_20_: antibody count is decreasing in normal group. Even though the actual measurements from two platforms are negatively correlated, we can construct the test statistics *t*_1_ and *t*_2_ so that the positive value of the statistics supports the alternative hypotheses and the weighted average can be used as combined evidence of the involvement of the biomarker in the process.

## Results

### Results on simulated data

In this section, we examine the performance of our proposed method by examining its positive selection rates and false discovery rates under various testing scenarios. We simulate data sets from *Q* different platforms. The number *Q* is set to be either 2 or 5. For the *q*th experiment, the data set is denoted as *X*_*q*_. For each data set, we assume that *n* different biomarkers are measured, *X*_*q*_ = ${\left(X_{q1}^{\prime },\ldots ,X_{\mathrm{qn}}^{\prime }\right)}^{\prime }$. For the *i*th biomarker, *X*_*qi*_= ${\left(X_{\mathrm{qi}1}^{\prime },X_{\mathrm{qi}2}^{\prime }\right)}^{\prime }$, where *X*_*qi*1_ denotes data from the control group with mean *μ*_*qi*1_ and *X*_*qi*2_ denotes data from the diseased group with mean *μ*_*qi*2_. The total number of biomarkers is set to be *n* = 1000. Among the *n* biomarkers, let *g* denote the number of biomarkers that are related to the biological process of interest, i.e. *μ*_*qi*1_ ≠ *μ*_*qi*2_. The number *g* of differentially expressed (DE) biomarkers is set to be 200. The number of measurements for each biomarker obtained from each platform is set to be 10, in which 5 are from the control group and the other 5 are from the disease group. We also consider different effect sizes. For continuous data, we generate $X_{\mathrm{qi}}∼\mathrm{MVN}\left({\left(\mu_{\mathrm{qi}1}^{\prime },\mu_{\mathrm{qi}2}^{\prime }\right)}^{\prime },\Sigma \right)$, where Σ has an exchangeable correlation structure with correlation *ρ*. The correlation *ρ* is set to be either 0 or 0.5. For differentially expressed markers, *μ*_*qi*1_ = 0 × 1_*m*_, *μ*_*qi*2_ = *e* × 1_*m*_, where *e* is the effect size and *m* = 5 is number of measurements. Discrete data *X*_*qi*_is generated from a Poisson(*λ*) distribution, where *λ*_*qi*1_ = *μ*_*qi*1_ for the control group and *μ*_*qi*2_ = *μ*_*qi*1_ + *e*for the diseased group. The *g* differentially expressed markers are divided into two groups with *g*_1_ = 100 and *g*_2_ = 100. Each group is assigned a different effect size *e*. For each platform, the alternative hypothesis can be either left-sided, right-sided or two-sided. The number of permutation is 100. All of the permuted values from the *n* biomarkers are pooled together to form the empirical null distribution. The results are summarized for 100 simulated data sets.

To compare our multi-platform integration method with the individual platform analysis method, the positive selection rate (PSR) and false discovery rate (FDR) are calculated to assess the performance of each method for selecting the differentially expressed biomarkers:

$$\text{PSR}=\frac{\text{\# of correctly identified DE biomarkers}}{\text{\# of DE biomarkers}}$$

and

$$\text{FDR}=\frac{\text{\# of falsely identified DE biomarkers}}{\text{\# of identified DE biomarkers}}$$

Tables 1, 2, and 3 provide detailed simulation settings and results at the *α* = 0.05 significance level. From the results, we can see that our multi-platform integration method has the highest PSR and the lowest FDR with the smallest variance compared to all other individual platform analyses in all scenarios. In addition, such advantage is consistently observed regardless of whether or not there is correlation among the measurements obtained for each biomarkers. Table 1 summarizes the results for the integrative analysis based on two different platforms. Given different effect sizes, different sided alternatives, and different correlations, the increase in PSR is consistently about 40% and the decrease in FDR is about 30% compared to the results from individual platforms. Table 2 summarizes the results for the integrative analysis based on five different platforms. Given different simulation scenarios, the increase in PSR for most cases is about 60% and the decrease in FDR is about 40% compared to the results from individual platforms. This shows that by integrating more data from different sources, we are improving the sensitivity and selectivity of the proposed method. Table 3 summarizes the results for the integrative analysis based on two different platforms, where the first consists of continuous data and the second consists of discrete data. Similar to the setting with two continuous data sets, the increase in PSR is about 40% and the decrease in FDR is about 30% compared to the results from individual platforms.

**Table 1:** The simulation settings and results for two platforms with continuous data

			**Methods**	
		**Multi-platform**	**1st individual**	**2nd individual**
Scenario 1:	*ρ* = 0; g = *g*_1_ + *g*_2_ = 200			
Right-side	Experiment1:	e = 0.5 for *g*_1_ = 100; e = 2 for *g*_2_ = 100		
	Experiment2:	e = 1.5 for *g*_1_ = 100; e = 1 for *g*_2_ = 100		
	*PSR Mean*	0.7895	0.5372	0.5588
	*PSR Var*	0.0007	0.0007	0.0010
	*FDR Mean*	0.1907	0.2680	0.2600
	*FDR Var*	0.0007	0.0013	0.0009
Left-side	Experiment1:	e = -0.5 for *g*_1_ = 100; e = -2 for *g*_2_ = 100		
	Experiment2:	e = -1.5 for *g*_1_ = 100; e = -1 for *g*_2_ = 100		
	*PSR Mean*	0.7908	0.5330	0.5556
	*PSR Var*	0.0006	0.0006	0.0012
	*FDR Mean*	0.1891	0.2673	0.2649
	*FDR Var*	0.0006	0.0009	0.0011
Two-sided	Experiment1:	e = -1 for *g*_1_ = 100; e = 1.5 for *g*_2_ = 100		
	Experiment2:	e = 2 for *g*_1_ = 100; e = -1 for *g*_2_ = 100		
	*PSR Mean*	0.6988	0.4113	0.5403
	*PSR Var*	0.0011	0.0011	0.0010
	*FDR Mean*	0.2145	0.3202	0.2694
	*FDR Var*	0.0007	0.0016	0.0012
Scenario 2:	*ρ*=0.5; g = *g*_1_ + *g*_2_ = 200			
Right-side	Experiment1:	e = 0.5 for *g*_1_ = 100; e = 2 for *g*_2_ = 100		
	Experiment2:	e = 1.5 for *g*_1_ = 100; e = 1 for *g*_2_ = 100		
	*PSR Mean*	0.9405	0.6319	0.7819
	*PSR Var*	0.0003	0.0005	0.0007
	*FDR Mean*	0.1560	0.2410	0.2051
	*FDR Var*	0.0005	0.0009	0.0007
Left-side	Experiment1:	e = -0.5 for *g*_1_ = 100; e = -2 for *g*_2_ = 100		
	Experiment2:	e = -1.5 for *g*_1_ = 100; e = -1 for *g*_2_ = 100		
	*PSR Mean*	0.9400	0.6316	0.7871
	*PSR Var*	0.0002	0.0004	0.0006
	*FDR Mean*	0.1605	0.2419	0.2024
	*FDR Var*	0.0005	0.0007	0.0006
Two-sided	Experiment1:	e = -1 for *g*_1_ = 100; e = 1.5 for *g*_2_ = 100		
	Experiment2:	e = 2 for *g*_1_ = 100; e = -1 for *g*_2_ = 100		
	*PSR Mean*	0.9377	0.6670	0.7327
	*PSR Var*	0.0003	0.0010	0.0007
	*FDR Mean*	0.1622	0.2270	0.2122
	*FDR Var*	0.0005	0.0009	0.0007

**Table 2:** The simulation settings and results for five platforms with continuous data

**Method**	**Multi-plat**	**1st ind.**	**2nd ind.**	**3rd ind.**	**4th ind.**	**5th ind.**
Scenario 1:	*ρ* = 0; g = *g*_1_ + *g*_2_ = 200					
	Exp1:	e = 1.5 for g = 200				
	Exp2:	e = 1.5 for *g*_1_ = 100; e = 1 for *g*_2_ = 100				
	Exp3:	e = -0.5 for *g*_1_ = 100; e = -2 for *g*_2_ = 100				
	Exp4:	e = -1 for *g*_1_ = 100; e = 1.5 for *g*_2_ = 100				
	Exp5:	e = 2 for *g*_1_ = 100; e = -1 for *g*_2_ = 100				
*PSRMean*	0.9517	0.5601	0.4130	0.4464	0.4213	0.4471
*PSRVar*	0.0002	0.0012	0.0011	0.0004	0.0010	0.0005
*FDRMean*	0.1572	0.2605	0.3299	0.3108	0.3205	0.2727
*FDRVar*	0.0004	0.0011	0.0018	0.0009	0.0010	0.0010
Scenario 2:	*ρ* = 0.5; g = *g*_1_ + *g*_2_ = 200					
	Exp1:	e = 1.5 for g = 200				
	Exp2:	e = 1.5 for *g*_1_ = 100; e = 1 for *g*_2_ = 100				
	Exp3:	e = -0.5 for *g*_1_ = 100; e = -2 for *g*_2_ = 100				
	Exp4:	e = -1 for *g*_1_ = 100; e = 1.5 for *g*_2_ = 100				
	Exp5:	e = 2 for *g*_1_ = 100; e = -1 for *g*_2_ = 100				
*PSRMean*	0.9998	0.8360	0.6655	0.5682	0.6712	0.5699
*PSRVar*	2.7e-06	0.0006	0.0010	0.0004	0.0010	0.0008
*FDRMean*	0.1281	0.1898	0.2217	0.2593	0.2314	0.2093
*FDRVar*	0.0004	0.0006	0.0009	0.0007	0.0007	0.0008

**Table 3:** The simulation settings and results for two platforms with continuous data and discrete data

		**Methods**	
	**Multi-platform**	**1st individual**	**2nd individual**
Experiment1:	Continues; *ρ* = 0; e = 0.5 for *g*_1_ = 100; e = 2 for *g*_2_ = 100		
Experiment2:	Discrete; *μ*_*qn*1_ = 5, e = 3 for g = 200		
*PSR Mean*	0.7356	0.5327	0.5228
*PSR Var*	0.0008	0.0004	0.0012
*FDR Mean*	0.1967	0.2702	0.2763
*FDR Var*	0.0008	0.0012	0.0012

**Figure 1:**
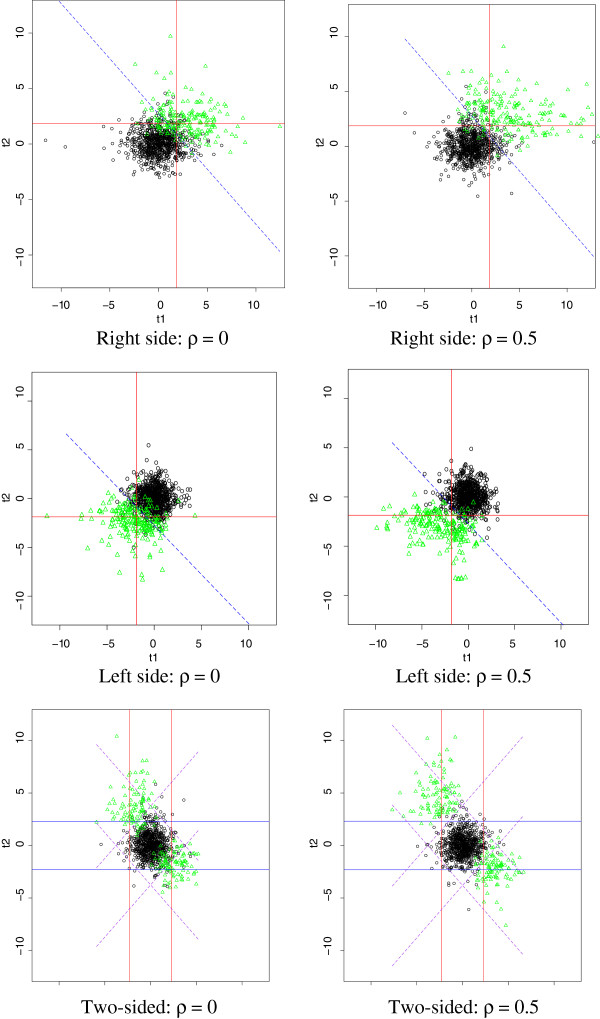
**Decision lines for comparing methods.** Vertical lines use data from the first individual platform, horizontal lines use data from the second individual platform, and dashed lines use our multi-platform integration method. Circles represent non-differentially expressed biomarkers and triangles represent differentially expressed biomarkers. Plots are based on one simulated data set and 100 permutations.

Figure 1 demonstrates decision lines from different methods. The plot is constructed based on the results from one simulated data set and contains three decision lines: the vertical line using data from the first individual platform, the horizontal line using data from the second individual platform, and the dashed line based on our multi-platform integration method. Our decision line provides a greatly improved separation of the differentially and non-differentially expressed biomarkers. Moreover, the individual platform analysis misidentifies some of the data points compared to our method.

As we examine a large number of biomarkers, we need to investigate the control of the false discovery rate of the proposed method with regards to multiple hypothesis testing [13]. Given a fixed cut-off value of *α*, we obtain the realized false discovery rate $\mathrm{FDR}=\left(\mathrm{FP}\right)/\left(\hat{T}P\right)$) and its estimates $F\hat{D}R=\left(\hat{F}P\right)/\left(\hat{T}P\right)$, where *FP* denotes the number of false positive biomarkers, $\hat{F}P=n\pi \alpha$ is the estimated number of false positive biomarkers, $\hat{T}P$ is the total number of biomarkers claimed as positive, *π* is the proportion of non-differentially expressed genes, and $\hat{\pi }$ is its estimator. We can control the estimated number of false positive discoveries by selecting the significance level of the approaches. We expect that the estimated $\hat{F}P$ should be close to the true *FP*; the $F\hat{D}R$ should be close to the true *FDR* as well. Under the simulation setting of scenario 2 left-sided case in Table 1, the control of the false discovery rate of our proposed method under different significance levels is examined and presented in Table 4. With *π* = 0.8 and *α* = 0.005, $\hat{F}P$ is aimed to be controlled at 4. On average, our method produces 3.84 false positives, whereas the first and second individual platform analyses has 4.65 and 5.00 false positives, respectively. The corresponding average $F\hat{D}R$ of our method is 0.0225, which is close to the true *FDR* of 0.0214. This demonstrates the integrative analysis yields satisfactory control of false discovery rate, which is improved compared to individual platform analyses.

**Table 4:** True positives and false discovery rates with *π* = 0.8

**Methods**	***α***	**0.05**	**0.01**	**0.005**
	$\hat{F}P$	**40**	**8**	**4**
multi-platform	$\hat{T}P$	224	165	143
	(*std*)	6.5547	6.0820	5.5202
	*FP*	44.8125	8.0250	3.8375
	(*std*)	7.3348	3.4778	2.263
	*FDR*	0.1563	0.0386	0.0214
	(*std*)	0.0219	0.0161	0.0125
	$F\hat{D}R$	0.1428	0.0388	0.0225
	(*std*)	0.0041	0.0014	0.0009
1st individual	$\hat{T}P$	165	107	91
	(*std*)	8.8797	5.3066	4.9031
	*FP*	50.5125	9.9000	4.6500
	(*std*)	8.9101	3.4982	2.1766
	*FDR*	0.2431	0.0736	0.0406
	(*std*)	0.0326	0.0246	0.0183
	$F\hat{D}R$	0.1940	0.0600	0.0353
	(*std*)	0.0103	0.0030	0.0019
2nd individual	$\hat{T}P$	197	106	79
	(*std*)	7.2442	8.2303	6.3222
	*FP*	48.9250	9.6000	5.000
	(*std*)	7.1862	3.5750	2.5376
	*FDR*	0.1986	0.0721	0.0506
	(*std*)	0.0245	0.0258	0.0251
	$F\hat{D}R$	0.1630	0.0607	0.0408
	(*std*)	0.0060	0.0048	0.0033

### Results on real data

In this section, we apply our method to data from a study of growth and stationary phase adaption in *Streptomyces coelicolor* provided by Jayapal et al. [16]. The data set contains both isobaric stable isotope labeled peptide (iTRAQ^*TM*^)-derived shotgun proteomic data and DNA microarray transcriptome data. To study different growth stages of *S. coelicolor* M145 cells, eight time point cell samples (7, 11, 14, 16, 22, 26, 34, and 38 h) were collected. Because the iTRQA^*TM*^ system can only analyze four distinct samples in a single experiment, the eight protein samples were distributed across three runs of mass spectrometric (MS) analysis, The protein sample from 11 h was run in three MS experiments, so it serves as a reference. Therefore, protein abundance ratios $r_{j/11\mathrm{hr},k}^{i}$ were obtained from experimental run *k* for protein *i* in sample *j*hr with respect to the 11 h reference. Protein identification and quantification were carried out by comparing the raw spectral data against a theoretical proteome of *S. coelicolor* using proteinPilot^*TM*^ software and the inbuilt Paragon^*TM*^ search engine. Only proteins identified with ≥ 99*%* confidence were considered for further analysis. Finally, all identified proteins were further processed to yield a protein abundance ratio with respect to the first time point (7 h) sample using $r_{j/7\mathrm{hr}}^{i}=r_{j/11\mathrm{hr}}^{i}/r_{7\mathrm{hr}/11\mathrm{hr}}^{i}$. Ultimately, only 886 proteins identified in the 7 h sample could be used for our analysis.

For microarray data, total mRNA from the same eight time point samples were isolated and a spotted DNA microarray experiment was conducted. Hybridization was performed using genomic DNA (gDNA) as a reference. The mRNA abundance was obtained using _log2_[cDNA/gDNA]. To be consistent with the protein data, mRNA abundance data from different samples were processed to calculate _log2_[cDNAi/cDNA_7*hr*_] for each sample with respect to the first time point sample. Only gene expression values with protein values (894 genes) were analyzed. To deal with missing values, we deleted genes that had no values for mRNA at all or had at least five missing values in the protein data set. The rest of the missing values for genes were imputed by using R package MICE. In total, the number of genes suitable for the subsequent integrative analysis was 886. Based on the growth curve, time points were divided into two groups; those from 7, 11, 14 and 16 h represented the growth phase and those from 22, 26, 34 and 38 h represented the stationary phase.

**Figure 2:**
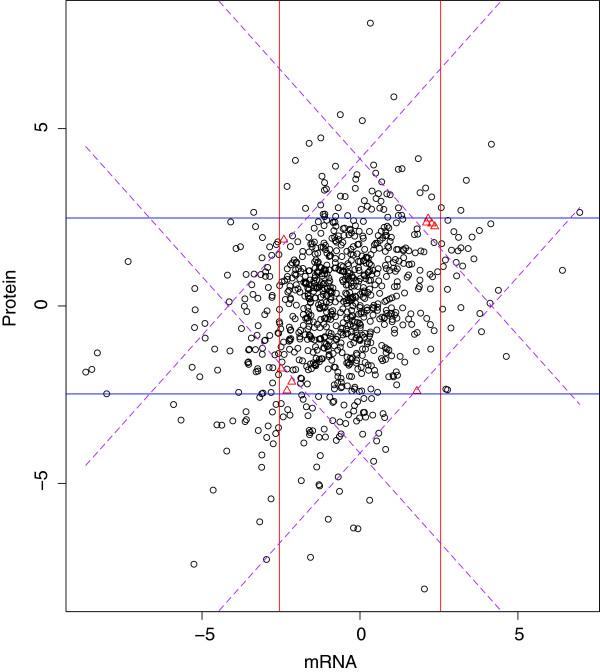
**Decision lines for real data.** Vertical lines use the mRNA data, horizontal lines use the protein data, and dashed lines use our multi-platform integration method.

The objective of our analysis is now to select the biomarkers that are differentially expressed between the two phases. We apply our multi-platform integration method to identify differentially expressed biomarkers. For the mRNA data, we formulate the null hypothesis as *H*_0_: the mRNA expression level is the same between the two phases. Similarly, for protein data, the null hypothesis is formulated as *H*_0_ : the protein ratio is the same between the two phases. For both mRNA data and protein data, two-sided alternatives are considered in the analysis. For each platform, we use Student’s t-statistics to summarize the statistical evidence, which are denoted as *t*_*m*_ and *t*_*p*_. To obtain the multivariate null distribution, 100 permutations are conducted. The overall correlation between *t*_*m*_ and *t*_*p*_ is 0.2787. The variances of *t*_*m*_ and *t*_*p*_ are 3.0489 and 3.6411, respectively. Based on the decision line constructed at the significance level *α* = 0.05, our method detects 172 differential expressed genes with an estimated $\hat{F}P$ equal to 44. Individual analysis on the mRNA data and the protein data detects 137 and 143 genes, respectively. Figure 2 depicts the decision lines for all three comparative analyses: the vertical lines using the mRNA data, the horizontal lines using the protein data, and the dashed lines using our multi-platform integration method.

Nine differentially expressed genes are identified by our method but not by the other two methods. Among these, we identify biosynthetic enzymes (SCO5080 actVA5, SCO5072 actVIORFI) involved in actinorhodin production. These genes are up-regulated only at late stages of the culture and produce antibiotics during the stationary phase. Expression of two genes encoding malate oxidoreductase (SCO2951) and translation elongation factor G (SCO4661) have been found to be depressed during the stationary phase compared with the growth phase [17]. Table 5 summarizes the nine genes and the associated literature confirmations [16-21].

**Table 5:** SCO Summaries for the 9 genes which are identified by multi-platform integration method but not by individual platform analysis

**SCO**	**Sanger**	**Sanger**	**Sanger**	**Sanger**	**TIGR**	**Related**
	**abbreviation**	**annotation**	**category**	**subcategory**	**category**	paper*
SCO1958	uvrA	ABC excision	Macromolecule	DNA-replication,	excinuclease ABC,	[17]
		nuclease subunit A	metabolism	repair, restr./modific’n	A subunit	[17]
SCO2940	other	putative	Not classified	Not classified	xanthine	
		oxidoreductase	(included putative	(included putative	dehydrogenase,	
			assignments)	assignments)	putative	
SCO2951	other	putative malate	Central intermediary	Other central	malate	[16,17,19]
		oxidoreductase	metabolisms	intermediary metabolism	oxidoreductase	
SCO3094	other	conserved	hypothetical	Conserved in	conserved	
		hypothetical	protein	organism other than	hypothetical	
		protein	protein	Escherichia coli	protein	
SCO4661	fusA	elongation	Macromolecule	Proteins -	translation	[16,17,19]
		factor G	metabolism	translation and	elongation	
				modification	factor G	
SCO5072	actVIORF1	hydroxylacyl-CoA	Secondary	PKS	hydroxylacyl-CoA	[16,17,20]
		dehydrogenase	metabolism	PKS	dehydrogenase	
SCO5080	actVA5	putative	Secondary	PKS	putative	[17,18]
		hydrolase	metabolism	PKS	hydrolase	
SCO6219	Other	putative ATP/GTP	Protein	Serine/		[17]
		binding protein,	kinases	threonine		
		putative serine				
SCO6222	other	putative	Not classified	Not classified	aminotransferase,	[15,17]
		aminotransferase	(included putative	(included putative	class I	
			assignments)	assignments)		

## Discussion

An ongoing problem in proteomics is that extremely small sample sizes often occur, largely due to biological reasons. To investigate the performance of our method in such situations, we consider a case for each platform wherein the control and the diseased groups each have only two measurements. Our method is applied and the simulation results shown in Table 6, scenario 1. Due to the small sample size, the positive selection rate is rather low and the false discovery rate rather high. Nevertheless, the combined method still outperforms the single platform method.

**Table 6:** Additional simulations

**Method**	**Multi-plat**	**1st ind.**	**2nd ind.**
Scenario 1:	Extremely small sample size		
	two measurements from each group		
*PSR Mean*	0.3022	0.2363	0.2179
*PSR Var*	0.0009	0.0006	0.0007
*FDR Mean*	0.3782	0.4436	0.4694
*FDR Var*	0.0023	0.0025	0.0027
Scenario 2:	Correlation among platforms set to 0.5		
	Disease and normal groups are independent		
*PSR Mean*	0.6689	0.5365	0.5578
*PSR Var*	0.0009	0.0008	0.0011
*FDR Mean*	0.2255	0.2690	0.2641
*FDR Var*	0.0008	0.0010	0.0010
Scenario 3:	Non-standardized version of *t*_*m*_ and *t*_*p*_		
	i.e. *t*_*m*_ = $\bar{x_{2}}-\bar{x_{1}}$, *t*_*p*_ = $\bar{y_{2}}-\bar{y_{1}}$		
*PSR Mean*	0.8142	0.5479	0.5992
*PSR Var*	0.0009	0.0005	0.0010
*FDR Mean*	0.1586	0.2358	0.2235
*FDR Var*	0.0006	0.0011	0.0010

We also consider the situation in which data on the same biomarker from *n* platforms have a multivariate distribution and the data from the diseased group are independent of those from the control group. The new simulation results are summarized in Table 6, scenario 2. The correlation between the platforms is set to 0.5, and the other parameters are the same as in Table 1, scenario 1, right-sided test. Due to the high correlation among the platforms, the gain in power of the aggregated method is less pronounced than that of the independence case. This is because different platforms contribute overlapping information when they are highly correlated.

The proposed method allows different ways of constructing *t*_*m*_ and *t*_*p*_ as long as they provide summarized statistical evidence for that platform. The Student’s *t*-statistic is adopted in the paper simply for illustration purpose. Alternatively, we can simply use the unstandardized differences: $t_{m}={\bar{x}}_{1}-{\bar{x}}_{2}$, and $t_{p}={\bar{y}}_{1}-{\bar{y}}_{2}$. Then we proceed with the randomization, obtain the estimated variances for *t*_*m*_ and *t*_*p*_ and form a weighted linear sum statistic. To compare the empirical performance of the standardized versus unstandardized versions, we conduct simulations under the setting 1 of Table 1 with right-sided test. The results are summarized in Table 6, scenario 3. The two versions have comparable performance in terms of PSR and FDR. The unstandardized version of *t*_*m*_ and *t*_*p*_ has a slightly higher PSR and a slightly lower FDR.

An alternative way of combining test statistics across different platforms is to form a multivariate quadratic statistic. Given two platforms, for example, we consider an alternative test statistic

$$t_{Q}={\left(t_{m},t_{p}\right)}^{\prime }{\overset{ˆ}{\Sigma }}^{-1}\left(t_{m},t_{p}\right),$$

where $\hat{\Sigma }$ is the estimated covariance matrix of the vector (*t*_*m*_,*t*_*p*_) obtained from the empirical null distribution. Such multivariate statistic can be used to test the overall null hypothesis against two-sided alternatives, while the weighted linear statistic that we propose can be used to test one-sided alternatives or two-sided alternatives. Thus, our method is more broadly applicable. We further conduct simulations to compare the multivariate quadratic form with our proposed weighted linear statistic for two-sided tests under the setting of scenario 2, Table 1, with results included in Table 7. For two-sided alternatives, the quadratic statistic has very similar performance to our proposed weighted linear statistic, with a slightly lower PSR and a slightly higher FDR.

**Table 7:** Comparison with the quadratic test statistic *t*_*Q*_

**Method**	**Multi-plat**	**Quadratic**
*PSR Mean*	0.9377	0.9155
*PSR Var*	0.0003	0.0004
*FDR Mean*	0.1622	0.1804
*FDR Var*	0.0005	0.0005
Quadratic:	Exp1:	e = -1 for *g*_1_ = 100; e = 1.5 for *g*_2_ = 100
	Exp2:	e = 2 for *g*_1_ = 100; e = -1 for *g*_2_ = 100

Finally, we compare our method with the existing robust rank aggregation method [14] with results included in Table 8. The inference from rank aggregation method is based on the ranks of the test statistics. The ranking can in some degree reflect the significance of the test statistics. But the position of the rank does not always translate into the relatedness of the biomarker to the underlying biological mechanism. The rank aggregation method assigns p-values of the observed ranks under the null hypothesis that the normalized ranks of all biomarkers are uniformly distributed. But this is a null hypothesis which can correspond to two totally different situations: all the biomarkers are not related to the biological process or all of them are related with equal effect size. This evaluation of p-values under such global null hypothesis has two implications. First of all, if all the biomarkers are related to the biological process with equal or similar effect sizes, the observed ranks will appear non-informative and thus the method will have little power to detect them. Secondly, the p-value of each observed rank is calculated under the global null hypothesis. Thus, the rank aggregation has a correct error control under the global null hypothesis but has no correct error control under other configurations of the individual hypotheses. In other words, it lack the strong control of the error rate under different configurations of the individual hypothesis [15]. On the other hand, our method assigns p-values under the individual null hypotheses and thus have a strong control of the error rate. This means our method’s actual false discovery rate and estimated false discovery rate will be in good agreement no matter how many of the genes belong to the null situation and how many belong to the alternative situation. While in contrast, the rank aggregation will tend to be very conservative if there are many biomarkers belonging to the alternative situation. To demonstrate this, we choose the number of significant markers ranging from 100, 200 to 400. It is shown in Table 8 that the rank aggregation behaves very conservatively in the presence of large number of significant markers. For instance, with five platforms and 200 significant biomarkers, our proposed method has a PSR of 0.9995 and a FDR of 0.1399, while the competing rank aggregation method has a much lower PSR of 0.4995 and FDR of 0.0823. This comparison further demonstrates the advantage of the proposed method.

**Table 8:** Comparison with Robust Rank Aggregation Method

	**Setting:**	**Method**	**Multi-plat**	**RRA**
1.	*ρ* = 0.5; g = *g*_1_ + *g*_2_ = 100			
	Exp1: e = 1.5 for g = 200	*PSR Mean*	1.000	0.7497
	Exp2: e = 1.5 for *g*_1_ = 100; e = 1 for *g*_2_ = 100	*PSR Var*	1.98e-6	0.0012
	Exp3: e = -0.5 for *g*_1_ = 100; e = -2 for *g*_2_ = 100	*FDR Mean*	0.2803	0.0912
	Exp4: e = -1 for *g*_1_ = 100; e = 1.5 for *g*_2_ = 100	*FDR Var*	0.0011	0.0003
	Exp5: e = 2 for *g*_1_ = 100; e = -1 for *g*_2_ = 100			
2.	*ρ* = 0.5; g = *g*_1_ + *g*_2_ = 200			
	Exp1: e = 1.5 for g = 100	*PSR Mean*	0.9995	0.4995
	Exp2: e = 1.5 for *g*_1_ = 50; e = 1 for *g*_2_ = 50	*PSR Var*	0.23e-06	0.0008
	Exp3: e = -0.5 for *g*_1_ = 50; e = -2 for *g*_2_ = 50	*FDR Mean*	0.1399	0.0823
	Exp4: e = -1 for *g*_1_ = 50; e = 1.5 for *g*_2_ = 50	*FDR Var*	0.0004	0.0004
	Exp5: e = 2 for *g*_1_ = 50; e = -1 for *g*_2_ = 50			
3.	*ρ* = 0.5; g = *g*_1_ + *g*_2_ = 400			
	Exp1: e = 1.5 for g = 100	*PSR Mean*	0.9992	0.1133
	Exp2: e = 1.5 for *g*_1_ = 50; e = 1 for *g*_2_ = 50	*PSR Var*	2.23e-6	0.0002
	Exp3: e = -0.5 for *g*_1_ = 50; e = -2 for *g*_2_ = 50	*FDR Mean*	0.0402	0.0796
	Exp4: e = -1 for *g*_1_ = 50; e = 1.5 for *g*_2_ = 50	*FDR Var*	0.0001	0.0015
	Exp5: e = 2 for *g*_1_ = 50; e = -1 for *g*_2_ = 50			

## Conclusion

With the advent of various types of genomic technologies, it is imperative to develop a method that can integrate different types of genomic data to solve biological questions. We develop a general framework for data integration across multiple data platforms. For each data set, a test statistic is formed to summarize the statistic evidence toward the specific null hypothesis tailored to the data platform. The types of test statistics can vary and their marginal distributions can be different. The observed test statistics can then be aggregated across different data platforms. The overall decision is based on the empirical distribution of the aggregated statistic obtained through random permutations. Our method can accommodate different experimental designs and various data types across platforms.

## Competing interests

The authors declare that they have no competing interests.

## Authors’ contributions

SW, XG, YX, XW and ZF developed the algorithm, SW and YX implemented the algorithm, YX, ZF, and XY performed data analysis; and XG supervised the project. All authors read and approved the final manuscript.
